# A cluster-randomized, placebo-controlled, maternal vitamin a or beta-carotene supplementation trial in bangladesh: design and methods

**DOI:** 10.1186/1745-6215-12-102

**Published:** 2011-04-21

**Authors:** Alain B Labrique, Parul Christian, Rolf DW Klemm, Mahbubur Rashid, Abu Ahmed Shamim, Allan Massie, Kerry Schulze, Andre Hackman, Keith P West

**Affiliations:** 1 Center for Human Nutrition, Department of International Health, Bloomberg School of Public Health, Johns Hopkins University, Baltimore, Maryland, USA; 2 The JiVitA Maternal and Child Health and Nutrition Research Project, Gaibandha, Bangladesh

## Abstract

**Background:**

We present the design, methods and population characteristics of a large community trial that assessed the efficacy of a weekly supplement containing vitamin A or beta-carotene, at recommended dietary levels, in reducing maternal mortality from early gestation through 12 weeks postpartum. We identify challenges faced and report solutions in implementing an intervention trial under low-resource, rural conditions, including the importance of population choice in promoting generalizability, maintaining rigorous data quality control to reduce inter- and intra- worker variation, and optimizing efficiencies in information and resources flow from and to the field.

**Methods:**

This trial was a double-masked, cluster-randomized, dual intervention, placebo-controlled trial in a contiguous rural area of ~435 sq km with a population of ~650,000 in Gaibandha and Rangpur Districts of Northwestern Bangladesh. Approximately 120,000 married women of reproductive age underwent 5-weekly home surveillance, of whom ~60,000 were detected as pregnant, enrolled into the trial and gave birth to ~44,000 live-born infants. Upon enrollment, at ~ 9 weeks' gestation, pregnant women received a weekly oral supplement containing vitamin A (7000 ug retinol equivalents (RE)), beta-carotene (42 mg, or ~7000 ug RE) or a placebo through 12 weeks postpartum, according to prior randomized allocation of their cluster of residence. Systems described include enlistment and 5-weekly home surveillance for pregnancy based on menstrual history and urine testing, weekly supervised supplementation, periodic risk factor interviews, maternal and infant vital outcome monitoring, birth defect surveillance and clinical/biochemical substudies.

**Results:**

The primary outcome was pregnancy-related mortality assessed for 3 months following parturition. Secondary outcomes included fetal loss due to miscarriage or stillbirth, infant mortality under three months of age, maternal obstetric and infectious morbidity, infant infectious morbidity, maternal and infant micronutrient status, fetal and infant growth and prematurity, external birth defects and postnatal infant growth to 3 months of age.

**Conclusion:**

Aspects of study site selection and its "resonance" with national and rural qualities of Bangladesh, the trial's design, methods and allocation group comparability achieved by randomization, field procedures and innovative approaches to solving challenges in trial conduct are described and discussed. This trial is registered with http://Clinicaltrials.gov as protocol NCT00198822.

## Background

Community trials, incorporating principles and methods of a randomized, controlled design, represent a "gold standard" methodology for generating evidence on efficacy or effectiveness of health and nutrition interventions. In low-income countries, where resources may be minimal, infrastructure undeveloped and political stability uncertain, numerous challenges exist to their design and conduct. A study site should reflect, to the degrees possible, numerous characteristics of the population for whom results are to be interpreted, qualities that can promote generalizability of future findings. For trials seeking to measure impact on rare events, such as mortality, study size, reflected in sample size and numbers of data handling staff, and duration, which may extend for years, present challenges to maintaining data accuracy and reliability. Large and long trials may be subject to substantial size- and time-dependent biases that can occur with procedural drift, in the absence of adequate, organized, supervised and enforced quality control. Beyond meeting technical demands, scientific rigor and ethical obligations, operational success of a large community trial requires a well developed, multifaceted and adequately funded organization, supportive political and social relationships, and sensitivity toward minimizing distortions to the local economy and culture.

Over the past decade, large field trials have been conducted to evaluate the efficacy of antenatal micronutrient supplement use on maternal and infant mortality, in Southern Asia [1,2] and Africa [3-6], helping to establish levels of impact of practical interventions under diverse environmental, nutrition and disease conditions. While informative, there remains a general lack of substantive descriptions and critical treatises on design, site, and methodological and organizational requirements for implementing large, community based nutrition trials. This paper seeks to help fill this gap by describing aspects of design, study site selection, technical challenges encountered and solutions developed to maintain quality control and optimize efficiencies during the conduct of a large (60,000 pregnancy/44,000 infant), long (6.5 year), cluster-randomized, controlled trial in rural northern Bangladesh that evaluated the prophylactic efficacy of maternal vitamin A (VA) supplementation during and after pregnancy in reducing maternal, fetal and infant mortality. The trial was carried out in the rural Districts of Gaibandha and Rangpur, amidst a region historically affected by high maternal mortality, VA deficiency and poor health care [7].

## Methods

### Aims of the study

The study, called "JiVitA-1" represents the first large community trial undertaken by the JiVitA Health and Nutrition Research Project (JiVitA) from 2001 to 2007. The primary aim of JiVitA-1 was to establish the efficacy of a weekly oral dose of VA or beta-carotene, administered from early pregnancy through three months post-partum, at concentrations that approximated a recommended dietary allowance, in reducing all-cause mortality of women related to pregnancy by at least 35%. Secondary aims included determining the impact of maternal VA or beta-carotene supplementation during pregnancy and postpartum periods on fetal loss due to miscarriage or stillbirth, infant mortality under three months of age, maternal obstetric and infectious morbidity, infant infectious morbidity, maternal and infant micronutrient status, fetal and infant growth and prematurity, gross external birth defects and infant growth to 3 months of age.

### Study Site Selection

**Figure 1:**
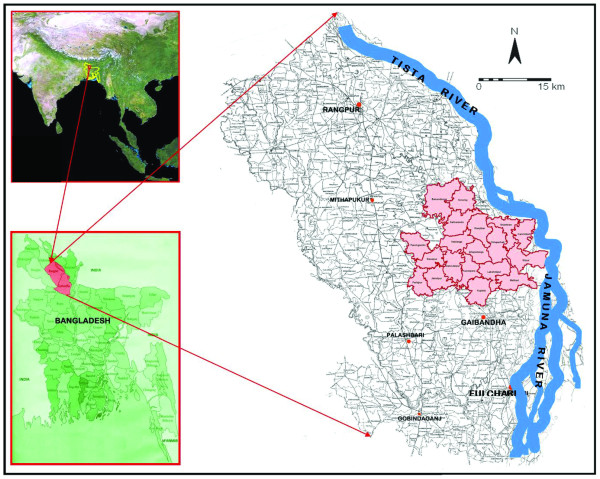
**The JiVitA Research Project setting, a population dense (~1000 persons per sq km), contiguous rural area of ~ 450 sq km at 30 m above mean sea level, west of the confluence of the Teesta and Jamuna rivers of Northwest Bangladesh**.

In order to support a large, cluster-randomized trial intended to inform national policy considerations, it was critical that the study site broadly reflect vital, health and nutritional risks and exposures of the national rural population, a quality of research sites we refer to as "resonance". The process of site selection involved several months of site visits throughout the country and abstraction of disaggregated national data from multiple sources on rural infrastructure, health care, agriculture and demography. An area comprising 19 rural unions in the northwest Districts of Gaibandha and Rangpur became the preferred site, based on survey reports of maternal night blindness, reflecting VA deficiency, remoteness and rural quality (e.g., mostly villages surrounded by rice fields, linked by unpaved roads), area size (~435 sq km) and contiguity, population density (~1000 per sq km) [7], agrarian nature (e.g., seasonality and crop mix, weekly market network), and a receptive political culture and administrative system,. These qualities were further supported by a relatively accessible, urban center (Rangpur City) lying within a few hours of the field site that could support data management and logistics requirements (e.g., electrical supply, telecommunications, trunk road access, basic residence facilities) (Figure 1) [8]. The area appeared to represent, broadly, the ~35^th ^percentile of economic and quality of life in rural Bangladesh, based on comparative reports of food production, flood risks and other environmental hazards, maternal and child diet and nutritional and health status and health care utilization [7], reflecting resonance with the national rural context of Bangladesh, as detailed in Table 1[9,10].

**Table 1:** Population characteristics of the study area (Gaibandha - column 3) compared to national statistics for Bangladesh as a whole (column 1), and only Rural Bangladesh (column 2).

	Bangladesh - Overall	Rural Bangladesh	Gaibandha	Difference between Rural Bangladesh and Gaibandha
	(1)	(2)	(3)	(2)-(3)
	%	%	%	%
***Women of Reproductive Age***				
**Age**, years				
15-19	21.9	21.8	20.3	1.5
20-24	18.1	18.1	19.9	-1.8
25-29	16.0	15.9	15.8	0.1
30-34	13.4	13.6	13.4	0.2
35-39	12.7	12.5	13.3	-0.8
40-44	9.5	9.5	10.0	-0.5
45-49	8.5	8.8	7.2	1.6
**Education**				
None	34.1	37.6	41.1	-3.5
Primary incomplete	13.8	15.5	12.3	3.2
Primary complete	11.9	12.5	9.0	3.5
Secondary incomplete	27.1	26.8	25.2	1.6
Secondary complete or higher	12.8	7.3	11.7	-4.4
Non-standard curriculum	0.4	0.4	0.7	-0.3

***Household characteristics***				
**Floor construction**				
Earth/sand	78.2	90.4	84.4	6.0
Palm/bamboo/wood	1.2	1.2	0.0	1.2
Cement	20.0	8.3	15.6	-7.3
**Roof construction**				
Thatch/leaf	6.8	7.8	4.7	3.1
Palm/bamboo	0.5	0.5	0.5	0.0
Metal/Tin	83.4	87.5	91.9	-4.4
Cement	7.5	2.2	2.9	-0.7
**Wall construction**				
Cane/palm/leaf/jute sticks	8.5	11.0	12.7	-1.7
Dirt/mud	15.5	17.9	15.7	2.2
Bamboo/Bamboo with mud	16.8	16.9	10.6	6.3
Tin sheet	34.9	39.2	43.0	-3.8
Bricks	21.2	12.0	16.7	-4.7

***Household assets***				
Electricity	50.5	39.3	37.5	1.8
Radio	30.2	29.3	27.7	1.6
Television	34.6	24.8	25.5	-0.7
Mobile phone	25.2	18.4	15.1	3.3
Watch	72.9	69.5	58.9	10.6
Bicycle	27.7	28.6	30.3	-1.7
Motorcycle or scooter	3.6	2.4	3.9	-1.5
Rickshaw van	5.7	5.5	6.5	-1.0
				

***Government Health Services***				
**Nutrition Program Coverage**				
Vitamin A supplementation - Children^1^	89.2	88.3	90.8	-2.5
Postpartum Vitamin A supplementation^2^	17.2	15.8	18.0	-2.2
**Child and Maternal Vaccination**^3^				
BCG	97.0	96.7	98.4	-1.7
DPT (1^st ^Dose)	96.6	96.2	98.4	-2.2
Polio (1^st ^Dose)	99.1	99.0	100.0	1.0
Measles	87.5	87.2	93.7	-6.5
Maternal tetanus toxoid (TT) ^4^	89.6	88.9	85.7	3.2

***Environmental characteristics***				
**Source of drinking water**				
Piped into dwelling or common area	6.6	0.3	2.0	-1.7
Tubewell/borehole	88.7	95.9	94.8	1.1
**Sanitation**				
Piped sewer system	2.9	0.2	0.3	-0.1
Septic tank system	13.2	7.2	15.7	-8.5
Closed-slab pit latrine (Sealed)	23.1	24.5	23.9	0.6
Open pit/Hanging Toilet	52.1	58.3	35.5	22.8
No facilities or bush or field	7.5	9.2	23.3	-14.1
Use of improved sanitation^5^	39.2	31.9	39.9	-8.0

***Reproductive Health***				
**Antenatal Care Provider**				
Medical doctor	37.1	31.1	26.3	4.8
Nurse/midwife	10.6	10.1	12.0	-1.9
Community health worker	4.6	5.1	5.3	-0.2
No ANC received	43.8	49.2	48.9	0.3
**Assistance at delivery**				
Medical doctor	15.5	10.6	7.5	3.1
Nurse/midwife	4.6	3.4	6.0	-2.6
TBA	66.0	71.1	48.1	23.0
Relative/Friend	11.2	12.3	33.1	-20.8
**Place of delivery**				
Home	82.7	88.2	89.2	1.0
Delivery in health facility^6^	16.0	10.7	9.0	1.7

***Education***				
**School attendance, by Sex**				
Primary school attendance (Male)	78.9	79.0	73.4	5.6
Primary school attendance (Female)	83.7	84.2	75.5	8.7
Secondary school attendance (Male)	36.2	33.6	38.3	-4.7
Secondary school attendance (Female)	41.4	39.4	36.3	3.1
**Adult (15-24 years) literacy**				
Female	69.9	67.6	62.8	4.8
***Early marriage***				
Married before age 15^7^	33.1	36.2	40.0	-3.8
Married before age 18^8^	74.0	78.4	79.7	-1.3
Married women, aged 15-19 years	41.9	46.1	51.3	-5.2

Public-access data analyzed from the 2006 Multiple Indicator Cluster Survey (MICS), conducted by the Bangladesh Bureau of Statistics (BBS) between June and October 2006, sponsored by UNICEF (16). A total 62,463 households (1,950 clusters) were surveyed, where 1,280 clusters were from rural areas and 38 were from Gaibandha (16). (Some category totals do not add to 100% as rows may have been omitted for brevity, or lack of information.)

^1^Children of age 9-59 months who received vitamin A supplementation in the last six months prior to the survey interview.

^2^Mothers aged 15-49 years with a birth in the two years preceding the survey who received high-dose vitamin A supplementation before the infant was 8 weeks old.

^3^Children aged 12-23 months with proof of current vaccination against childhood disease.

^4^TT: Mothers with live births in one year preceding the survey who were given at least two doses of tetanus toxoid vaccine within the appropriate interval prior to giving birth.

^5^Improved sanitation facilities are: flush to piped sewer system, flush to septic tank, flush to pit latrine and pit latrine with slab.

^6^Health facility: public sector and private sector health facilities.

^7^Numerator: number of women that were first married by the exact age of 15 and denominator: total number of women aged 15-49 years. ^8^Numerator: number of women that were first married by the exact age of 18.2 and denominator: total number of women aged 15-49 years.

### Field Organization

The trial required a rural addressing and surveillance system capable of identifying, enrolling and tracking large numbers of pregnant women, From March to December 2000, over 120,000 households in the study area were located and placed on digitized land-allocation maps as part of a JiVitA Geographic Information System (GIS), described elsewhere [11]. The study area was segregated into 596 units of both work management and randomization, called "sectors", each of ~250 households in size and covered by a single resident field worker (Field Supplement Distributor, or FD). A team of 10-12 FD's was supervised by a single Team Leader (TL), also a local resident and based in a local office. Four leaders of adjacent team areas (~40-44 sectors) were supervised by a single "Area Coordinator (AC)", also residing in the local area. The 12 coordinated areas were overseen by two field managers under direct administrative and technical and administrative direction of a senior management team and study investigators.

### Study Area Census

In July 2001, a study census enumerated 102,771 women eligible to participate in the JiVitA-1 trial. All consenting women of reproductive age (13-44 years), married and living with their husbands, not sterilized or menopausal or whose husbands were not sterilized, were eligible to be enlisted into a 5-weekly, home-based, pregnancy surveillance system. Pregnant or post partum, lactationally amenorrheic women were placed on a 'waiting list', and only became eligible for pregnancy surveillance once their menses resumed. Women entering the study area within 4 months of marriage were also eligible to join the cohort under pregnancy surveillance. Eligible pregnant women were enrolled over a period that began in August 2001 and ended in October 2006.

### Intervention

**Figure 2:**
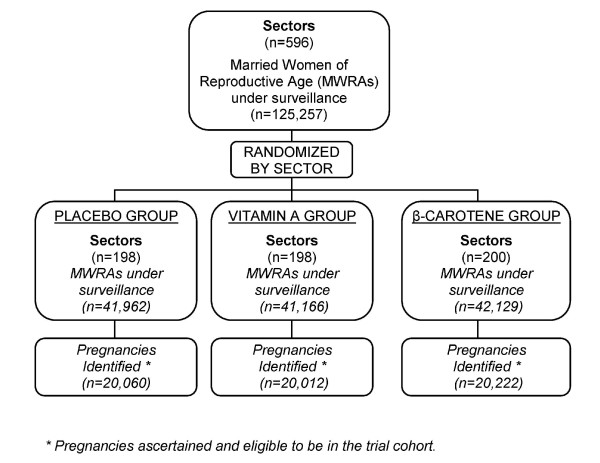
**Basic trial design of JiVitA-1 and pregnancy identification**.

Each woman under active surveillance was visited once every 5 weeks by the FD assigned to their sector. Women were asked about their menses in the past 30 days; if amenstrual, they were offered a urine-based hCG pregnancy test (Orchid Biomedical Systems, Goa, India). The 5-week interval was chosen as a standard length of time (with biologic variability) between pregnancy surveillance visits to allow normal menarcheal cycles to occur (28 ± 4 days)[12]. A shorter interval would have increased the risk of re-visiting subjects before menstrual onset, resulting in unnecessary pregnancy testing. Following a positive result, consenting pregnant women were administered a coded supplement each week, through 3 months postpartum. Ideally, supplements were taken under staff supervision, but when directly observed supplementation was not possible for a particular woman in a given week, a distinct code was recorded in the weekly dosing log. As the intervention was cluster-randomized, all enrolled pregnant women in a given sector received a weekly capsule containing one of the following masked ingredients: (1) Placebo (consisting of soybean oil with a small amount of vitamin E as an antioxidant), (2) VA (consisting of 7000 ug retinol equivalents, or 23,300 IU, of VA palmitate in soybean oil with a small amount of vitamin E as an antioxidant), or (3) Beta-carotene (consisting of 42 mg of *all-trans *beta-carotene, equivalent to 7000 ug of retinol equivalents, assuming a 6:1 conversion ratio). (See Figure 2).

### Outcomes

The primary outcome of interest was all-cause pregnancy-related mortality defined as death from any cause during pregnancy through 12-weeks post-pregnancy outcome (including early pregnancy losses such as miscarriages, induced and spontaneous abortions and stillbirths) per 100,000 pregnancies. Maternal vital status was assessed weekly as women continued to be visited by female supplement distributors through 3 months post partum. Family members of deceased women were visited by a trained research physician, who conducted a "Maternal Verbal Autopsy" interview.

Fetal loss due to miscarriage or stillbirth was defined as a pregnancy that ended without a live birth before 28 weeks or ≥28 gestation, respectively. Induced abortions, or 'menstrual regulations', were also registered as outcomes. Pregnancy losses were assessed weekly during supplementation visits to the home and confirmed with a pregnancy test following the reported loss. Women who experienced a pregnancy loss were visited within a week or two of the reported loss by a study interviewer who conducted a "Miscarriage/Stillbirth" interview.

Perinatal, neonatal and three-month mortality were defined as (a) stillbirth plus deaths within the first 7 days of birth, (b) infant deaths occurring within 28 days of a live birth, and (c) infant deaths occurring with the first 12 weeks of life, all per 1000 live births. Infant vital status was assessed weekly through 12 weeks of age by a study interviewer, and monthly thereafter. Parents of deceased infants were visited by a trained interviewer who conducted an "Infant Verbal Autopsy" interview.

Maternal obstetric and infectious morbidity was assessed by a structured interview conducted 3 months after pregnancy outcome and based on self-reported history of symptoms that occurred in the seven days before or after the pregnancy outcome, and in the 3 month period after the outcome. Any woman reporting night blindness in early pregnancy was given a "Night Blindness pictorial advice card" which depicted and described locally available beta-carotene and vitamin-A rich foods for consumption. At ~28 weeks of gestation, night blindness was again assessed by interview and suspected cases were offered the standard WHO regimen for treatment of night blindness in pregnancy (25,000 IU VA weekly for four weeks at a time, for a maximum of 12 weeks), irrespective of supplement allocation group.

At both three and six months of age, triplicate measurements of infant head, chest and mid-upper arm circumference were taken by field workers trained and standardized in anthropometry to assess infant growth. At these timepoints, in addition to infant and maternal morbidities described above, detailed information on breast and complementary feeding, vaccinations, possible congenital malformations, and antenatal and postpartum care was collected.

Prematurity was defined as a live birth occurring <37 weeks gestational age calculated using the interval between the woman's last menstrual period and the infant's date of birth.

### Sample Size

Restricting the number of eligible pregnancies to one per woman, the original sample size for the JiVitA-1 trial required to detect a ≥35% reduction in all-cause mortality in either supplement arm relative to control was calculated to be 54,000 (18,000 per group), allowing for a Type I error of 5%, power of 80%, equal supplement allocation, design effect resulting from the randomization of clusters rather than individuals of 21% (1.21), early pregnancy loss rate of 15% (12% miscarriage, 3% abortion) and loss to follow-up of 10%. The design effect used was based on measured outcomes from our earlier trial conducted in Nepal, and was not adjusted with data from the Bangladesh site [13]. The calculation assumed an estimated control group mortality rate of 800 per 100,000 pregnancies. The *a priori *comparisons of interests were between the placebo group and either active arm (VA or placebo). We considered the active arms to be comparable treatment groups, so their comparison (VA vs. beta carotene) was not of interest from a design point of view. Based on data collected during the first year of the trial, the sample size was increased, upon recommendation by the trial Data Safety Monitoring Board (DSMB), to 22,580 pregnancies per group, or a total of 67,740 assuming a re-estimated control group mortality rate of 500 per 100,000 pregnancies, a rate of pregnancy loss due to "menstrual regulation"/induced abortion of 25%, a Type I (α) error of 0.05, a Type II (β) error of 0.20, a design effect of 1.21 and a revised estimate of loss to follow-up of 2%. (Figure 2)

The sample size estimate per group specified above was also sufficient to detect a 15-20% reduction in either fetal loss or 3-month infant mortality, assuming control group rates of at least 50 deaths per 1000 pregnancies (for fetal loss) or per 1000 live births (for infant mortality), respectively, accepting the probability of an Type I error being 0.05 and a Type II error of 0.20, respectively.

### Cluster Randomization

The JiVitA-1 trial was designed as a cluster randomized trial, thus, clusters of individuals, rather than individuals, were randomly allocated for all consenting women of reproductive age within a given cluster to be given one of the three trial supplements throughout pregnancy until the end of three months post partum. Investigators sought to define clusters which would achieve the maximum number of clusters within the study population, while being sufficiently large to make up a working area for a field worker. The 'one treatment code per field worker' approach also addressed concerns about the possibility of inadvertent incorrect dosing if a single field worker were carrying three different masked supplements. Based on population density, terrain and the expected crude pregnancy rate in the study area (~31 per 1000 population), JiVitA "sectors" of approximately 250 households each were constructed with the expectation of generating, on average, 25-30 pregnancies per year.

**Figure 3:**
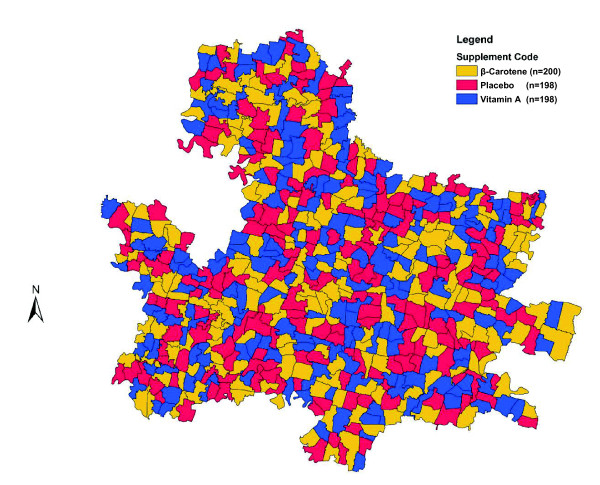
**Study area map illustrating the randomized distribution of treatment allocation to study sectors**.

**Figure 4:**
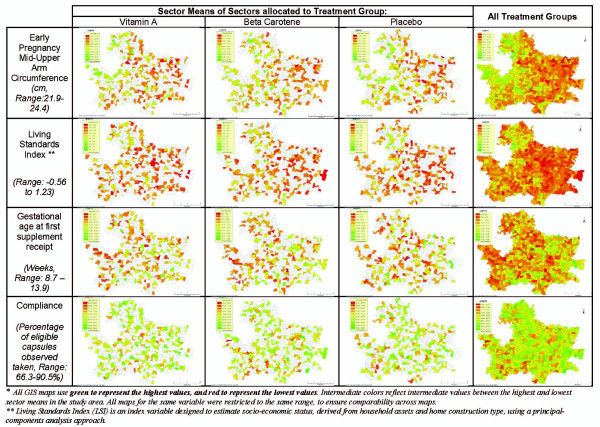
**GIS maps of the JiVitA-1 study area, illustrating successful sector-level randomization by displaying sector means of four select characteristics of enrolled women in those sectors (early pregnancy arm circumference, living standards index **[14]**, gestational age at first dose, and dosing compliance throughout the duration of the study)**.

In early 2001, the study area was divided into 596 smaller community groups of comparable size, with a mean±SD number of households of 231 ± 46 in each, which served as units of randomization (Figure 2). To randomize the intervention, all sectors were listed according to their 3-digit number ranging from 001 to 596. Sectors were randomized in blocks of nine, to one of three codes-1, 2 or 3. A block of 9 clusters was selected to create balance of supplement allocations within each supervisory area of 9-12 clusters. Three sets of 3 identical coins on which the numbers 1, 2 or 3 were written were placed into a container, mixed and removed randomly, without replacement, and the 3-digit code of each sector was read aloud sequentially. The code inscribed on the selected coin was assigned to that particular sector. We engaged field supervisors directly in the process of randomization, to increase the transparency of sector allocation among workers and the local community. Table 2&3 and Figures 3 &4 display the sector-level and geo-spatial balance achieved by the randomization process, respectively.

**Table 2:** Sector-Level Characteristics, by treatment group

Presence in Sector	Placebo N = 198		Vitamin A N = 198		β-Carotene N = 200	
	n	%	n	n	n	%
**Market Structures**						
Permanent market **	15	7.6	40	40	28	14.1
Regular (weekly) market	15	7.6	19	19	10	5.1
Rice Mill *	112	56.6	134	134	107	54.0

**Education**						
Primary School	83	41.9	93	93	95	48.0
High School *	15	7.6	33	33	30	15.2

**Health Services**						
Private Doctor (MBBS)	2	1.0	6	6	6	3.0
Family Welfare Clinic *	6	3.0	16	16	7	3.5
Medicine Shop	39	19.7	57	57	50	25.2
Village Doctor	89	45.0	94	94	107	54.0

**Infrastructure**						
Year Round Finished Road	62	31.3	48	48	53	26.8
Electricity	99	50.0	103	103	94	47.5

* Significant differences between treatment groups, p-value <0.05

** Significant differences between treatment groups, p-value <0.01

**Table 3:** Household Socioeconomic Characteristics at Enrollment, by treatment group^1^

Baseline Socioeconomic Characteristics	PL N = 19,862		VA N = 19,806		BC N = 19,998	
	n	%	n	%	n	%
**Literacy**						
Read/write (self)	8473	42.7	8308	42.0	8451	42.3
Read/write (husband)	9079	45.9	8969	45.5	9026	45.4

**Woman's Education**						
Class 1 to Class 9	8469	42.7	8382	42.4	8445	42.4
Class 10 complete (SSC)	604	3.0	535	2.7	618	3.1
Class 11+	756	3.8	745	3.8	790	4.0

**Husband's Education**						
Class 1 to Class 9	6063	32.0	5969	31.6	5934	31.2
Class 10 complete (SSC)	863	4.6	819	4.3	845	4.4
Class 11+	1733	9.1	1760	9.3	1826	9.6

**Husband's Occupation**						
Farmer	5739	28.9	5662	28.6	5711	28.6
Laborer	5026	25.4	5025	25.4	5020	25.2
Fisherman	148	0.8	129	0.7	182	0.9
Contracted laborer	215	1.1	201	1.0	209	1.0
Business	6704	33.8	6747	34.1	6862	34.4
Private	1403	7.1	1438	7.3	1386	6.9
Government service	286	1.4	263	1.3	277	1.4
Other	304	1.5	301	1.5	310	1.6

Religion*						
Islam	18374	92.7	18404	93.1	18205	91.2
Hindu	1425	7.2	1350	6.8	1691	8.5
Other	25	0.1	20	0.1	64	0.3

**Household Assets**						
Own Cattle	7944	40.1	7822	39.6	7936	39.8
Own Goats/Sheep	4456	22.5	4500	22.8	4427	22.2
Own Bicycle	7486	37.8	7487	37.9	7491	37.5
Own Land	16462	83.5	16460	83.6	16438	82.9

Own Irrigation Pumps	2031	10.2	2039	10.3	2097	10.5
Have Electricity	2372	12.0	2469	12.5	2513	12.6

^1^Missing numbers (maximum number missing) 886, 917 and 963 for group PL, VA and BC, respectively.

* Significant differences between groups, p-value <0.001

### Intervention Masking

Study supplements were identical in color, taste and external appearance. Supplements were originally shipped in identically labeled 100-count white-opaque plastic bottles distinguished only by the code number-1, 2 or 3-listed on the label. Treatment codes were assigned by the Nutrilite Health Institute and were kept in a sealed envelope in a locked cabinet at both Nutrilite and Johns Hopkins University. Masking was further ensured by having a senior administrative staff (not involved in field activities) recode the supplement bottles with sector-specific permanent stickers bearing codes from 001 to 596. Bottles were distributed, as needed by field staff, using a controlled first-in first-out (FIFO) system, from a temperature and humidity-controlled and monitored, locked storeroom. Study participants, interviewers, field supervisors and investigators remained masked to treatment assignments until the end of the trial.

Supplements were released to the field, as needed, to replenish those consumed by the study participants. Supplement potency was regularly conducted every 6 months by independent testing laboratories and new batches of supplements were produced every 2 to 2.5 years, depending on stock. Supplement potency results were regularly made available to the trial DSMB and ranged from 93.6% to 120.6% for beta-carotene and to 67.7 to 114% for VA.

### Clinical and Biochemical Substudies

One year after the start of the main trial, a contiguous area of 32 study clusters, balanced across treatment allocations, was identified as a "Substudy Area". Approximately 600 mother-infant pairs from each treatment group, for a total of 1800 women, representing a ~3% sample of the overall study, were followed more intensively for changes in micronutrient status in order to document the efficacy of supplementation. This sample was estimated to be adequate to detect expected changes between supplemented and the placebo groups of 8-10 μg/dl in serum retinol concentration with a Type I error of 0.05 and a Type II error of 0.20. Consenting, newly pregnant women underwent additional clinical, biochemical, body compositional, and infectious disease protocols that included home visits during 1^st ^and 3^nd ^trimester of pregnancy and at 3 months post-partum. Women were measured for weight (SECA Red Line Solar Scale, UNICEF), height (Harpenden Pocket Stadiometer, Shorr Productions, MD, USA), mid-upper arm circumference (adapted from the Zerfas insertion tape) and triceps and sub-scapular skin-folds (Holtain Ltd, UK) during the pregnancy visits. In addition, venous blood and a vaginal swab (self-administered) were collected and women were measured for single-frequency bioelectrical impedance (RJL Systems, MI, USA) and blood pressure. A sample of urine was also collected and tested for sugar and/or protein content using a dipstick method (URiSCAN, Yeongdong Diagnostics, Korea), after which about 10 ml of remaining urine was banked at -20°C. Infants born to mothers in this sample were also measured for size at birth and at 3 months of age including weight (SECA Red Line Solar Scale, UNICEF), length (Length board, Shorr Productions, MD, USA), and head, chest and arm circumference at each visit. At a 3 month postpartum visit, maternal anthropometry was again collected (weight, MUAC and triceps skin-fold) and breast milk, maternal (venous) and infant (heelstick) blood was collected.

Laboratory analysis was performed at the Institute of Nutrition, Mahidol University (Bangkok, Thailand) to assess VA, carotenoid, and vitamin E concentrations in plasma and breastmilk. Ferritin and other nutritional assays in plasma were performed at the micronutrient laboratory at the Center for Human Nutrition (Johns Hopkins Bloomberg School of Public Health, Baltimore, MD, USA). Hemoglobin levels were measured at the time of each blood draw with a Hemocue hemoglobinometer (Hemocue, Inc., Mission Viejo, CA). Severely anemic women (Hb < 7 g/dl) were treated with 120 mg iron and 400 μg folate supplements for 3 months following WHO guidelines.

A birth defect surveillance system was established to monitor levels of visible birth malformations in each of the three study arms. Trained field workers conducted a complete external visual examination of all infants at their 3-month visit, noting any anomalies or deviations from normal, erring on the side of increased sensitivity and lower specificity. These anomalies were then checked by either a paramedic or physician, who performed a thorough examination of the child, documenting any anomaly with high-resolution digital photography. Photographs, maintained in a large image database, and examiner's notes were archived for future review by an expert teratologist to diagnose and classify any birth defects. Children identified with cleft lip and/or palate defects were offered free surgical correction through a local humanitarian organization.

### Ethical Approval

The study protocol was reviewed and approved by the Johns Hopkins Bloomberg School of Public Health Institutional Review Board (IRB) and the Bangladesh Medical Research Council (BMRC). Both institutional review boards periodically (annually, in the case of the Johns Hopkins IRB) reviewed the progress of the trial, wording, content and use of informed consent and assent procedures. The informed consent process included, when so desired by the subjects, husbands, in-laws and sometimes community members. Ultimately, individual consent was sought from the participating women and documented.

A Data Safety and Monitoring Board (DSMB) was formed to examine, over the course of the trial, its proper implementation and to monitor the safety and efficacy of interventions being tested. It included a total of 5 experts from Bangladesh (n = 2), the US (n = 2), and the United Kingdom (n = 1) with training and relevant expertise in reproductive health, public health nutrition, epidemiology, obstetrics and gynecology and biostatistics. The DSMB met four times throughout the study, when ~30%, ~55%, 70% and 85% of the planned sample size had been reached, to assess methodological adherence to the study protocol, quality control of data collection, compliance of enrolled women with supplement receipt, steps to protect human subjects, and evidence of benefit or harm. The JiVitA-1 study was registered with ClinicalTrials.gov as trial number ID GHS-A-00-03-00019-00.

## Results

### Major Field Procedures

#### Initial Census

**Figure 5:**
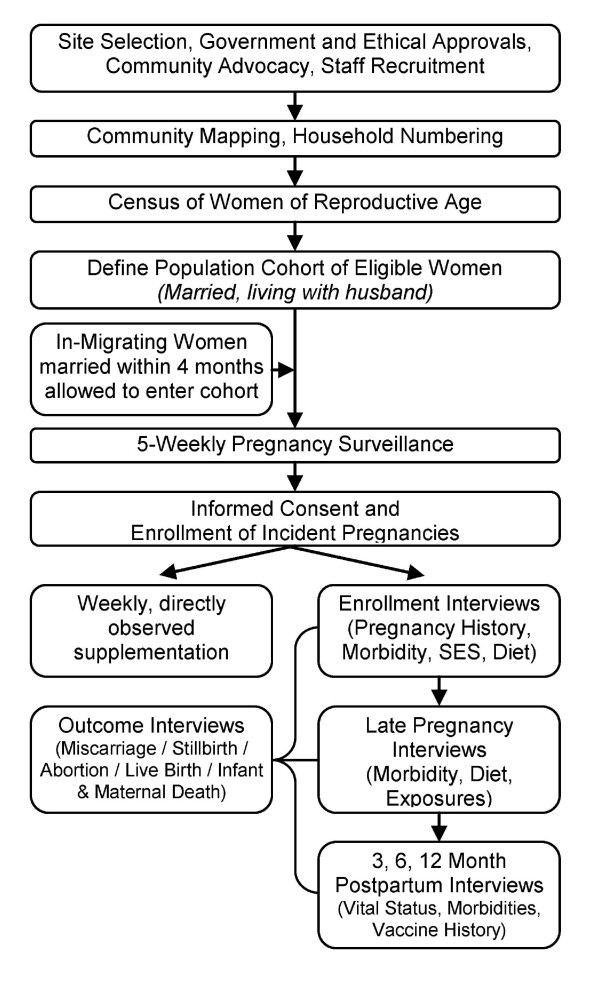
**Flow diagram of main JiVitA-1 trial procedures**.

From July-August 2001 a staff of 596 local female workers carried out a door-to-door census of every household in the study area to identify all women eligible to contribute a pregnancy to the trial. Women of reproductive age living with their husbands were listed on a roster that included the unique address of their house (Sector + household number) and a unique single digit number to distinguish multiple women in one household. This list was computerized and used to identify pregnant women during subsequent 5-weekly pregnancy surveillance rounds. Amenorrheic women who were either pregnant or breast feeding an infant under 12 months of age at the time of the initial census were placed on a "Waiting List" until they resumed menstruation or their infant turned 12 months of age, whereupon they were placed onto the pregnancy surveillance roster. (See Figure 5.) The schedule of surveillance, interviews and consent interactions are described below, and summarized in Table 4.

**Table 4:** Schedule of surveillance, interviews and consent interactions in the main JiVitA-1 trial

Survey/Questionnaire	Scheduled at	Contents	Conducted by *	Consent/Reconsent
Women's Enrollment Survey	Beginning of trial	Baseline census of all women of reproductive age, enrollment into surveillance	FD	Y
Newly Married Woman Survey	Ongoing, 5- weekly	Allows enrollment of eligible, newly married women	FD	Y
Pregnancy Surveillance Survey	Ongoing, 5- weekly	Urinary hCG-based identification of new pregnancies	FD	Y, for urine test
Women's Consent Interview	Pregnancy identification	Informing women about the trial, obtaining consent for interviews and supplementation	TL	Y
Pregnancy Dosing Visits	Weekly	Weekly supplementation of enrolled pregnant women	FD	N

Pregnancy Enrollment Questionnaire	First week after consent	Baseline characteristics, pregnancy history, 7 d diet, 7 d/30 d morbidity, exposures	FI	N
Socioeconomic Status Questionnaire	First week after consent	Education, employment of self and husband, household assets	FI	N
Family History Questionnaire	First week after consent	Parental vital status, cause of death, chronic morbidity	FI	N

Late Pregnancy Questionnaire	At 28 weeks gestation	7 d diet, 7 d/30 d morbidity, exposures	FI	N
Three-Month Postpartum Questionnaire	At 12 weeks postpartum	Complications of labor and delivery, 7 d diet, 7 d/30 d morbidity, exposures	FI	N
Six-Month Postpartum Questionnaire	At 24 weeks postpartum	7 d diet, 7 d/30 d morbidity, exposures	FI	N
Miscarriage/Stillbirth Questionnaire	4 weeks after event	Complications of labor and delivery, care-seeking	FI	Y
Verbal Autopsy - Infant	4 weeks after event	Enhanced from WHO standard instrument	FI	Y

Verbal Autopsy - Mother	4 weeks after event	Enhanced from WHO standard instrument	RP	Y

* FD = Field Distributor, must be local resident with minimum education level of 8^th ^grade TL = Team Leader, must be local resident with a minimum of a college degree

FI = Field Interviewer, must be local resident with a minimum of a college degree

RP = Research Physician, minimum MBBS-degree, with specific verbal autopsy training

At the outset, the surveillance roster contained the names of 102,771 women. The list was continuously updated as eligible, newly-married women became resident in the study area, as described earlier. Every 25 weeks, a list of women eligible for pregnancy surveillance, stratified by study sector, was printed and provided to field workers as a roster to visit women and screen for incident pregnancy.

#### Pregnancy Surveillance

The field trial formally began on August 12, 2001 with the initiation of pregnancy surveillance for the purpose of identifying and enrolling pregnant women into the trial. Using the roster, a team of nearly 600 field staff was responsible to perform pregnancy ascertainment every five weeks by asking women about the occurrence of their menstrual period in the previous month and offering a urine-based pregnancy test to those who reported not having menstruated.

#### Enrollment, Supplementation and Follow-up of Pregnant Women

Women with a positive pregnancy test were visited, typically within a week of the test, by a supervisor (team leader) who explained details of trial participation in a standardized fashion and sought consent to participate in the weekly supplementation trial and periodic home interviews. Irrespective of consent, all women were provided with a color graphical pamphlet entitled "Advice for Pregnant Women", designed by the Obstetric and Gynecologic Society, Bangladesh (OGSB), along with a maternal and infant immunization schedule. Consenting women were interviewed soon after by a trained field interviewer to obtain a pregnancy history, socioeconomic data, information about recent (past 7 and 30 day) morbidity symptoms, dietary and alcohol intake, tobacco product use and household chores performed in the previous week. At this time, maternal nutritional status was also assessed by measuring mid-upper arm circumference. Consenting women continued to be visited each week by local female supplement distributors during pregnancy through 12 weeks postpartum and given a coded capsule under staff supervision.

#### Night blindness assessment and treatment

At 28 weeks of gestation, following a pre-printed, sector-specific, 'night blindness assessment roster', FDs inquired about night blindness using an identified local term (*"ratkana/aloandari"*). Positive respondents were revisited within a week by a trained field interviewer who gathered additional symptoms to confirm the diagnosis. "Confirmed" cases were offered the WHO-recommended treatment for night blindness in pregnancy (25,000 IU VA every week for 4 weeks); if symptoms failed to resolve, subjects were provided 4 additional weeks of VA therapy, up to a maximum of 12 weeks of supplementation.

#### Infant registration

Live born infants were registered by the field distributors usually within a week of birth, during their routine supplementation visits to homes.

#### Three month post partum interview

A trained field interviewer returned to the home of participating women 12 weeks after the pregnancy outcome, irrespective of the type of outcome, and interviewed women to obtain (a) maternal re-consent for interview procedures, (b) maternal histories of illnesses during and following pregnancy, including detailed histories during the puerperal period (c) histories of infant breast and complementary feeding practices and morbidity symptoms, (d) to assess infant growth by circumferential measurements, and (e) to identify gross physical deformities by visual inspection. As described earlier, perceived physical abnormalities prompted a later visit and clinical examination by a trained research physician or paramedic, which included digital photography for future expert review. Histories of gross abnormalities for deceased infants were obtained by a verbal autopsy interview.

#### Cessation of maternal supplementation

At 12 weeks postpartum, participating mothers ceased being visited at home and supplemented on a weekly basis. Each study participant was eligible to contribute a single pregnancy to the JiVitA-1 trial. Each household continued to be visited, however, even after cessation of supplementation, on a five-weekly basis for the purposes of identifying any newly married women entering the study area.

#### Six-month post partum interview

Participating mothers were revisited and interviewed again to obtain histories of morbidity, diet, activity, infant feeding and morbidity, and to assess infant growth, employing the same procedures performed at the 3-month visit. Verbal autopsies were conducted for deceased infants.

#### Cause of Death Investigation (Maternal and Infant Verbal Autopsy)

Within 1 to 2 months following the death of an enrolled woman, a home visit was conducted by a trained interviewer to elicit a history of morbidity and other events that occurred or conditions that existed in the weeks leading up to death. Data collected from these "verbal autopsies" were independently reviewed by two physician reviewers (always including one obstetrician/gynecologist and one generalist), and a direct and underlying cause of death was assigned. Following the independent review, a joint consensus was reached on the most likely direct and underlying causes of death for each case.

Infant Verbal Autopsies were conducted soon after the infant's death, by a trained non-medical interviewer. Each infant "verbal autopsy" was independently reviewed by a team of two trained Research Physicians, and a consensus on the direct and underlying cause of death reached through discussion. When consensus could not be reached, a third independent review was performed.

#### Quality Control

Activities to ensure high-quality and standardized interview and measurement procedures included a rigorous recruitment process to select and hire qualified persons demonstrating aptitude for field work, provision of extensive training, certification of interviewers through standardization exercises, weekly supervisory visits of field activities by a two-tiered quality control system, a weekly peer-review of completed forms, outlier analysis and investigation of frequency distributions for questionnaire responses and anthropometric measurements, and random re-interviews conducted by a six-member quality control unit. Interviewers were also directly observed, and participants were visited randomly to evaluate the quality of the interaction with the interviewer. A computer-controlled roster was used to track field area visits and specific staff member visits to ensure all areas and staff were supervised with comparable intensity over the course of the trial. Regular quality control of all anthropometric instruments was performed, ranging from calibration of all equipment (scales, calipers, and stadiometers) on a daily basis, to a systematic, study-wide instrument calibration every three months.

#### Study Management

A series of weekly meetings were held every week at different levels of the field staff hierarchy which served as an information conduit between the field workers, field supervisors, and study managers up to the project investigators. Every Thursday, each team of field distributors (described earlier) met with their respective Team Leaders, who prepared a consolidated report of new pregnancies, pregnancy outcomes, and in-migrating, eligible married women of reproductive age. Any field problems, logistic needs, or procedural issues were raised and addressed at this meeting. Every Sunday morning, four Team Leaders met with their Area Coordinator, to convey their area's performance, review any new protocols or procedural changes and to exchange any data destined for the Data Management Center (DMC). On Sunday evenings, all the Area Coordinators (n = 14) converged at the project field headquarters for a full weekly debrief and interaction with the project management team, including scientific, logistic, financial, and administrative officers. Thus, on a weekly basis, a cycle of consecutive meetings served as an effective channel for primary data to flow from the field to the DMC (and vice versa), but also for information to flow, in both directions, to and from each one of over 850 field and project staff.

### Data Flow and Management

The data management system for the study included customized procedures covering a range of essential activities from tracking the transmittal of data from the field to the DMC, the entry and quality control of completed forms, the querying (where possible) of missing or invalid data, to the archiving of physical forms. Once collected, the data were reviewed and corrected, if necessary, at the field level, and forms packaged, grouped by type, into waterproof data folders, enumerated on a transmittal list by a supervisor, and sent to the DMC by project vehicle in locked data trunks every week. The transmittal list was checked upon arrival at the DMC against forms received and were then assigned to data entry operators (n = 12) for data entry. Missing forms could thus be immediately sought and recovered from the field with the help of the field supervisors.

Data entry was performed on networked PCs using Microsoft SQL Server (v8.0, Microsoft^®^, Seattle, WA, USA) as the relational database engine. The relational database was designed to maximize data efficiency and minimize database size through limited data redundancy. Data entry screens were custom-designed using Active Server Pages (ASP, Microsoft^®^, Seattle, WA, USA), which mimicked the paper forms through an HTML interface. Javascript code validated the entered data using standard range, consistency and logical checks within fields; logical consistency across multiple forms received for a particular participant was ensured via server-side checks against the SQL database.

Critical data such as identifiers were always entered twice. To quantify error rates by form, worker, and over time, and to further reduce data entry errors, each week a purposeful sample of forms was re-entered by a different data entry operator. A script flagged any discrepancies between the two entries; conflicts were investigated and resolved by a data supervisor. Individual data entry operator performance was tracked and specialized supervision, re-entry, or retraining was conducted as necessary. Data entry operators were also evaluated and trained in ergonomic positioning and required to take 10-minute breaks each hour to prevent eyestrain. An overall average error rate of less than 3 per 10,000 keystrokes was calculated for the JiVitA data entry team over the course of the study.

Problems identified during data entry or in data cleaning were logged for supervisor assessment. Some problems were resolved by review of the source data or of other related forms. Other problems requiring additional data from the field were assigned unique error numbers, described in detail on a printed form and sent to the appropriate field supervisor for investigation and resolution. Field queries were returned within an average of 2 weeks, and were actively managed in a query resolution database. Once resolved to the satisfaction of the Data Supervisor, the data was corrected on the source form and edited in the database. Although from the beginning, all modifications and corrections to the database were documented and archived systematically, from March 2003, a complete electronic record of every change to the database after initial data entry (including the time and person making the change) was preserved to prevent any undocumented data manipulation or erroneous data modification. The ongoing checking of the database helped improve the data quality by providing timely feedback and turn-around of error correction, and allowed for a more timely analysis at the end of the study.

Data were initially backed up daily onto rewriteable CD-ROMs, which were eventually replaced by portable external hard-drives. These were stored off-site in a locked, secure cabinet. An encrypted copy of the database was also uploaded to Johns Hopkins initially on a monthly basis, and as internet connectivity improved, on a weekly basis using a connection authenticated via RSA and encrypted using Advanced Encryption Standards (AES) over a Secure Sockets Layer (SSL) connection. The data entry terminal network was at all times isolated from the office network with internet access to eliminate any data security risks.

## Discussion

The conduct of large, population-based trials in remote, resource-poor settings represents substantial challenges to maintaining scientific integrity. The number of enrolled subjects needed to demonstrate impact on a rare event such as mortality (even in high risk areas) usually requires a sizeable population catchment area and a large and multifaceted team of field workers, supervisors, and technical, administrative and logistics support staff. It is often best to select workers by community transparent methods that identify skills and aptitude, though such staff usually arrive with little prior research experience. In JiVitA-1, detailed training and standardization protocols were developed to train cadres of field and support staff, including community surveillance workers, interviewers and supervisors, management, accounting, inventory and logistics staff. The layout and organization of field offices was carefully defined, from specific guidance on weekly meeting management and documentation, to the physical location of supplements, forms and other materiel. This enabled supervisors and workers to substitute for each other, as needed, across the large area, in case of medical or other leave. Another focus of JiVitA staff training was the standardization of handwritten English numbers (0-9) and letters (A-Z) that facilitated data entry and reduced error rates in data recording, coding and entry. We documented training curricula and required competencies for each level of worker that enabled consistency in performance over time, especially in the presence of occasional worker turnover.

As with any community-based activity, understanding the local culture and adopting appropriate approaches to obtaining informed consent are essential to fulfill ethical obligations to inform and assure volunteer involvement. Consent processes were adapted to the context, by engaging, as appropriate, the household heads, husbands, and mothers-in-law. Our procedures had to allow for an extended consent period as women often requested the opportunity to discuss their participation with their families prior to consent.

In this conservative, traditional and rural population, a number of strategies were adopted to reach, inform and communicate with our target, female and often pregnant and lactating audience - including the employment of a largely local, female workforce (>90% of our 850 staff), active engagement of the regional and local community leadership, and a good neighbor policy that involved assisting district communities, especially in times of emergency [8]. Advocacy and continuing education meetings engaged the formal and informal medical communities, the local government and health system, as well as grassroots NGO constituencies working in the area.

Most study activities depended on strictly prescribed information flow and activity 'cycles', ranging from the weekly supervisor-field worker meetings, to the regular, field-wide, quality control rounds. The cyclic flow of data from the field to the data management system, and the return of new information, instructions and queries back to the field in the form of pre-printed 'smart' rosters or forms allowed for increased efficiency and decreased opportunity for error by reducing handwriting and coding in the field. For example, by pre-filling the "End of Supplementation" boxes on supplementation rosters, we avoided worker calculation errors. We employed modern, but inexpensive barcoding to 'identify' pre-printed roster pages; when these pages returned with hand-written data, their entry was expedited significantly by a simple barcode scan. Preprinted data was recalled from a database and used to populate critical identifier, name and location fields - in under one second, compared to 3-5 minutes required to double-enter the same data.

Innovations in worker performance monitoring were facilitated by relatively easy access to data in modern relational databases. Data entry operators were individually graded on a monthly basis, based on entry rate, form-specific error frequency, and query generation. Field-based errors were documented in the database and used to provide feedback to field supervisors, by frequency of error, but without worker identifiers linked to specific faults (e.g., digit preferences in continuous measures) to avoid subsequent overcorrection and resultant biases in subsequent data collection by outlier staff. Using data-driven report software, we created 'anthropometry report cards' as quality control tools for supervisors to identify possible data falsification or procedural inconsistencies. These report cards calculated, over a specified period, measures of digit preference (unexpectedly high rates of certain terminal digits in anthropometric measurements, such as X.0 or X.5), rates of three identical measurements when taken in triplicate, and comparisons of individual median values, compared to global (all interviewer) medians.

Besides the primary study database, secondary management databases were created to systematize processes ranging from supplement and field logistic distribution to community and local health system advocacy contacts. The latter ensured frequent and regular courtesy visits to private and government doctors located across the study site, to address any concerns and to maintain a sense of community ownership of the ongoing trial. Each supervisory visit to one of 56 field managers was logged in a 'lightning strike' database, to fairly distribute unannounced quality control visits across the 435 sq. km. study area. This also served as a mechanism to monitor the supervisors themselves.

The weekly cycle of data transfer, supervisory meetings, and investigator feedback throughout the course of this trial allowed for rapid responses to queries from field workers, timely community management, and correction of data errors with minimum delay. At the field level, these cycles were introduced into every participating household through the provision of project-printed annual calendars. Workers marked their visits on these calendars, hung in a prominent place in each village home. Participants were encouraged to mark discretely the start date of their menstrual cycles on the calendar, further improving the recall of last menstrual period (LMP, for gestational age calculation) and outcome dates. These calendars were also used as supervisory tools as they documented the date and frequency of surveillance worker visits.

Finally, we continually explored novel applications of GIS and GPS technologies to enhance field operations beyond the core function of identifying and following study participants over time. As shown in Figure 4, the display and geospatial analysis of cluster-level data allowed us to demonstrate spatial balance in randomization, as well as identify real geographic differences within a fairly homogenous population (e.g. an east-west mid-upper arm circumference gradient; a socio-economic status gradient from northwest to southeast). This understanding of spatial variance is important in understanding possible inter-worker variability in anthropometric and socioeconomic data, even within a fairly homogenous rural setting. We also used real-time geographic plotting of refusals to Substudy activities (e.g. blood draws, antenatal checkups) as well as study-wide weekly rates of unmet subjects to identify unanticipated community reactions or poor worker performance, respectively. This permitted rapid, proactive early responses to these situations. Equipping field physicians with relatively inexpensive handheld GPS units, updated with the locations of scheduled interview households, increased their weekly work productivity significantly; an analysis of verbal autopsies conducted in the weeks prior to and following the introduction of GPSs revealed a 20% increase in weekly performance of these limited, highly trained project staff.

## Conclusion

In addition to the core research methods of the JiVitA-1 trial, these examples illustrate context-specific procedures, modifiable for other applications, that were developed to implement and manage a large community-based maternal supplementation trial. Their principal aim was to improve efficiency, quality and scientific validity of human subject contact and data collection during the trial. Continuous emphasis on innovation, especially adaptation of new technologies, while stringently adhering to accepted principles of trial conduct, can drive community-based research at scale to be cost-effective, time-appropriate and efficient. Low levels of resource represent challenges to overcome, but never excuses for failing, to achieve scientific rigor and credibility. The resonance of this northern, rural Gaibandha and Rangpur site with rural Bangladesh, and population settings across Gangetic South Asia, may help to promote uptake and application of findings from this and future JiVitA trials.

## List of Abbreviations

RE: Retinol Equivalents; UNICEF: United Nations Children's Fund; GIS: Geographic Information System; GPS: Global Positioning System; FD: Field supplement Distributor; TL: Team Leader; AC: Area Coordinator; hCG: Human Chorionic Gonadotropin; WHO: World Health Organization; VA: Vitamin A; ug: micrograms; IU: International Units; mg: milligrams; DSMB: Data Safety Monitoring Board; SD: Standard Deviation; FIFO: First-In First-Out; UK: United Kingdom; USA: United States of America; IRB: Institutional Review Board; BMRC: Bangladesh Medical Research Council; OGSB: Obstetric and Gynecologic Society, Bangladesh; SQL: Structured Query Language; PC: Personal Computer; HTML: HyperText Markup Language; CD-ROMs: Compact Disk Read Only Media; RSA: Rivest, Shamir and Adleman Security; AES: Advanced Encryption Standards; SSL: Secure Sockets Layer; NGO: Non-Governmental Organization.

## Competing interests

The authors declare that they have no competing interests.

## Authors' contributions

ABL was a lead coinvestigator for this trial and led the writing, editing and coordination of this manuscript, and oversaw the field implementation of the trial. PC was a lead coinvestigator for this trial and oversaw the design, implementation and analysis of this trial. RDWK was a lead coinvestigator for this trial and oversaw the design, implementation and analysis of this trial. Both PC and RDWK also oversaw the field training and QC/QA activities and helped to draft this manuscript. MR was a lead national co-investigator for this trial, and helped to design elements of this trial, and oversee the implementation of the trial protocol, in addition to contributing to the writing and editing of this manuscript. AAS, also a lead national coinvestigator, oversaw the quality control and training of the trial staff, and helped to manage the trial activities. AAS edited and commented on the manuscript. AM and AH served as the key technical, database management and programming leads for this trial, helping to write relevant aspects of the operational manual and this manuscript. KS oversaw and ran the micronutrient laboratory, including the transmittal, analysis and archival of biospecimens, and helped to edit the manuscript. KPW served as the principal investigator of this trial and is primarily responsible for the design, conduct and oversight of all aspects of the research, and contributed extensively to the writing of this manuscript. All authors read and approved the final version of the manuscript.
